# Renin–angiotensin system inhibitors reduce cardiovascular mortality in hypertensive patients with severe aortic stenosis undergoing transcatheter aortic valve implantation: insights from the EffecTAVI registry

**DOI:** 10.3389/fcvm.2023.1234368

**Published:** 2023-08-24

**Authors:** Christian Basile, Costantino Mancusi, Anna Franzone, Marisa Avvedimento, Luca Bardi, Domenico Angellotti, Domenico Simone Castiello, Andrea Mariani, Rachele Manzo, Nicola De Luca, Plinio Cirillo, Giovanni De Simone, Giovanni Esposito

**Affiliations:** Department of Advanced Biomedical Sciences, University of Naples “Federico II”, Naples, Italy

**Keywords:** TAVI, TAVR, aortic stenosis, renin–angiotensin system, ACE inhibitors, arterial hypertension

## Abstract

**Objectives:**

Arterial hypertension is associated with the triggering of the renin–angiotensin system, leading to left ventricle fibrosis and worse cardiovascular outcomes. In this study, patients with comorbid arterial hypertension and severe aortic stenosis (AS) undergoing transcatheter aortic valve implantation (TAVI) were selected from the EffecTAVI registry to evaluate the impact of angiotensin-converting enzyme inhibitors (ACEIs) or angiotensin II receptor blockers (ARBs) on cardiovascular mortality.

**Methods:**

We enrolled 327 patients undergoing TAVI from the EffecTAVI registry. Using Kaplan–Meier event rates and study-stratified multivariable Cox proportional hazards regression models, we evaluated 2-year clinical outcomes according to the ACEI/ARB therapy status at enrollment.

**Results:**

Among the included patients, 222 (67.9%) were on ACEIs/ARBs at baseline, whereas 105 (32.1%) were not. Treatment with ACEIs/ARBs was significantly associated with a 2-year decrease in the rate of cardiovascular mortality (HR = 0.44, 95% CI: 0.23–0.81, *p* = 0.009). This association remained stable after both multivariable adjustment and propensity score matching.

**Conclusion:**

In a cohort of hypertensive patients with severe AS who were selected from the EffecTAVI registry, ACEI/ARB treatment at baseline was found to be independently associated with a lower risk of 2-year cardiovascular mortality, suggesting a potential benefit of this treatment. More trials are needed to validate this finding and to understand the full benefit of this treatment.

## 1. Introduction

Excessive left ventricular (LV) mass and LV fibrosis are linked to poor outcomes in patients receiving transcatheter aortic valve implantation (TAVI) for severe aortic stenosis (AS) (1). Both LV hypertrophy and fibrosis result from total LV hemodynamic load, because the combination of the loads forced by valvular blockade and arterial burden (2) is mainly pressure overload. Excessive pressure burden on the LV is related to the activation of the renin–angiotensin-aldosterone system (RAAS), which is a direct mechanism for myocardial hypertrophy and fibrosis (3). Therefore, RAAS inhibitors such as angiotensin-converting enzyme inhibitors (ACEIs) and angiotensin II receptor blockers (ARBs) might reduce the burden of LV pressure in AS patients, which will have a favorable impact on LV remodeling to reduce hypertrophy and fibrosis (4). ACEIs/ARBs also reduce blood pressure (BP) levels, hence decreasing the overall LV hemodynamic burden (5). Until a few years ago, ACEIs/ARBs were considered potentially harmful and even contraindicated in severe AS because they could potentially cause a rapid and harmful decrease in BP levels (6). However, a recent meta-analysis that included observational and randomized evidence indicated that ACEIs/ARBs could be harmless and could even be helpful for treating AS (4, 7, 8).

In the light of the above, the aim of this analysis is to evaluate whether baseline, pre-TAVI treatment with ACEIs/ARBs influences cardiovascular outcomes in hypertensive patients with severe symptomatic AS undergoing TAVI.

## 2. Methods

### 2.1. Study design

Patients with severe AS were evaluated, as defined by a mean gradient >40 mmHg or a jet velocity >4.0 m/s, or an aortic valve area under 0.8 cm^2^ or 0.5 cm^2^/m^2^, and New York Heart Association (NYHA) Class II or higher. The exclusion criteria were chronic kidney disease (CKD) >stage III [Estimated Glomerular Filtration Rate (eGFR) < 30 ml/min/1.73 m^2^, computed with the CKD-EPI 2009 equation] or renal replacement therapy, significant aortic regurgitation, a LV ejection fraction lower than 20%, and predicted life expectancy <1 year. Clinical data, therapeutic information, electrocardiogram, and transthoracic echocardiograms were acquired at baseline during hospital admission and discharge and at 30 days, 3 months, 1 year, 18 months, and 2 years. Detailed medical history of the patients was recorded and clinical examination was performed on site by a renowned cardiologist. Auscultatory or oscillometric semiautomatic sphygmomanometers normally used by physicians were used for BP measurement, with cuffs of appropriate size. Systolic and diastolic BP were measured after a 5 min resting interval in the sitting position and three times at a 1 min interval in accordance with the current guidelines on hypertension (9). The average of the two last measurements was taken as the clinical BP. Standard transthoracic echocardiography was performed using a VIVID E95 ultrasound system (GE Healthcare) with a regular cardiological 3.5 MHz probe by following the recommendations of the European Society of Cardiology (10). Relative wall thickness was calculated as the ratio between posterior wall thickness and LV internal radius at end diastole and was considered high if it was ≥0.43. LV systolic function was assessed by using the LV ejection fraction (10). Electrocardiography was performed as a regular 12-lead rest electrocardiogram procedure.

The present analysis included only hypertensive patients with BP >140/90 mmHg or who were under antihypertensive medications.

Data were extracted from the EffecTAVI registry, an observational study that was designed to prospectively evaluate the safety and efficacy of the TAVI procedure and related clinical outcomes (registration number: NCT05235555, registered on 1 September 2015) (11). The study was approved by the Ethics Committee of the University of Naples Federico II.

### 2.2. Endpoints

The primary outcome of this study was cardiovascular mortality during follow-up. All patient deaths were considered cardiovascular deaths unless otherwise specified. Deaths that occurred during the performance of the TAVI procedure or before hospital discharge were not considered in the analysis.

### 2.3. Statistical analysis

Continuous variables were described as either mean ± standard deviation or median and interquartile range on the basis of the normality of distribution and compared by using Student's *t*-test or the Mann–Whitney test, respectively. Categorical variables were described as frequencies and percentages and compared by using Fisher's exact test or the *χ*^2^ test, as appropriate. Using the log-rank test, time-to-event variables were described and compared by using Kaplan–Meier event rates.

The multivariable Cox proportional hazards regression model was used to conduct the primary analysis. Covariates were included on the basis of the rule of one variable per 10 events. The included variables, other than age and sex, were those that were found to be more significant when the univariable analysis was performed, and those included in the adjusted models were age, sex, heart failure, and CKD (serum creatinine ≥2 mg/dl).

The propensity score model was developed using logistic regression, which modeled the likelihood of treatment with ACEIs/ARBs at baseline. This model included variables that significantly varied (*p* < 0.1) between patients who were on ACEIs/ARBs and those on other antihypertensive medications, and these variables were heart failure, sex, CKD, and atrial fibrillation. A 1:1 ratio propensity score matching was performed using a greedy nearest neighbor algorithm, which resulted in a propensity score–matched cohort comprising 210 patients—105 receiving ACEI/ARB therapy at baseline and 105 not receiving them. Standardized differences are reported when comparing baseline characteristics. To account for the matched nature of the data, the log-rank test and Cox proportional hazards regression were used to conduct survival analyses.

## 3. Results

### 3.1. Baseline characteristics of the population

This analysis included 327 patients with severe AS undergoing TAVI and treated for arterial hypertension. The baseline characteristics of the study population are presented in Table 1.

**Table 1:** Baseline population characteristics, divided by treatment with or without ACEIs/ARBs.

Variables	Patients not on ACEIs/ARBs (*N* = 105) *n*, %	Patients on ACEIs/ARBs (*N* = 222) *n*, %	*p*-Value
Sex Male (N/Y)	32 (30.5)	96 (43.3)	0.027
Age (mean ± SD)	83.48 ± 7.82	82.36 ± 6.57	0.18
Weight (kg; mean ± SD)	71 ± 17	72 ± 15	0.83
Body mass index (kg/m^2^; mean ± SD)	27 ± 6	28 ± 5	0.67
Systolic blood pressure (mmHg; mean ± SD)	128 ± 20	134 ± 20	0.014
Diastolic blood pressure (mmHg; mean ± SD)	71 ± 11	72 ± 11	0.3
Hemoglobin (g; mean ± SD)	13 ± 2	12 ± 2	0.5
Ejection fraction (mean ± SD)	53.8 ± 11.9	54.4 ± 11.4	0.67
Relative wall thickness (mean ± SD)	0.49 ± 0.10	0.48 ± 0.11	0.81
Aortic valve area (cm^2^)	0.46 ± 0.35	0.41 ± 0.34	0.24
Heart failure (N/Y)	38 (36.2)	57 (25.7)	0.051
Chronic kidney disease (N/Y)	45 (42.8)	57 (25.7)	0.002
Atrial fibrillation (N/Y)	40 (38)	43 (19.4)	<0.001
Diabetes (N/Y)	33 (31.4)	86 (38.8)	0.20
Dyslipidemia (N/Y)	70 (66.7)	153 (68.9)	0.68
Smoking (N/Y)	7 (6.7)	20 (9.0)	0.47
Coronary artery disease (N/Y)	48 (45.7)	97 (43.7)	0.73
Peripheral artery disease (N/Y)	43 (41)	104 (46.8)	0.32
Chronic obstructive pulmonary disease (N/Y)	33 (31.4)	67 (30.2)	0.82
Beta blockers (N/Y)	72 (68.6)	150 (67.6)	0.86
Alpha blockers (N/Y)	13 (12.4)	24 (10.8)	0.68
Diuretics (N/Y)	71 (67.6)	146 (65.8)	0.74
Calcium channel blockers (N/Y)	15 (14.3)	59 (26.6)	0.013
Antiplatelet therapy (N/Y)	57 (54.3)	152 (68.5)	0.013
NYHA class II (N/Y)	31 (29.5)	62 (27.9)	0.19
NYHA class III (N/Y)	54 (51.4)	110 (49.5)	0.21
NYHA class IV (N/Y)	20 (19.0)	50 (22.5)	0.31
eGFR (mean ± SD)	57.3 ± 25.0	63.2 ± 23.0	0.037

The patients were divided according to the use of ACEI/ARB therapy. Treatment with ACEIs/ARBs was associated with a higher systolic BP at baseline, male sex, and a higher prevalence of heart failure, atrial fibrillation, and CKD (all *p* < 0.05, Table 1).

During the 2-year follow-up, cardiovascular mortality occured in 43 patients.

**Figure 1:**
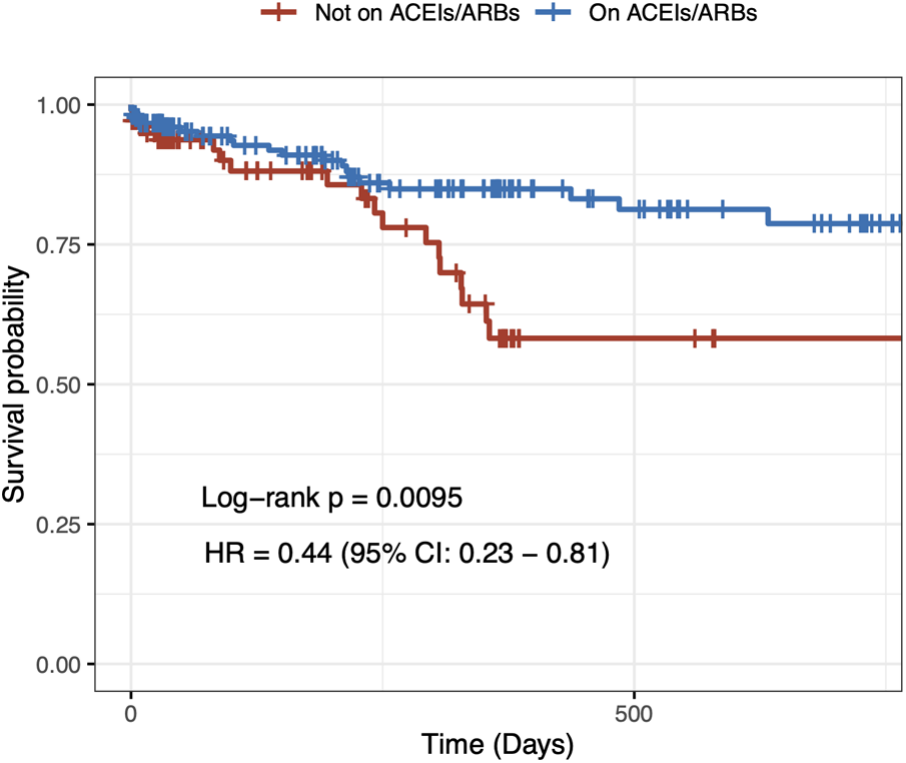
Two-year unadjusted cardiovascular mortality. Crude Kaplan–Meier survival curves according to angiotensin-converting enzyme inhibitor/angiotensin II receptor blocker treatment at baseline in hypertensive patients undergoing transcatheter aortic valve implantation.

In the univariable Cox regression analysis, it was found that the 2-year cardiovascular mortality rate was significantly lower in patients taking ACEIs/ARBs at baseline (HR = 0.44, 95% CI: 0.23–0.81 *p* = 0.009) (Figure 1). After a multivariable adjustment was made for age, sex, heart failure, and CKD, baseline treatment with ACEIs/ARBs remained associated with a decreased risk of cardiovascular mortality (HR = 0.51, 95% CI: 0.28–0.95 *p* = 0.034) (Table 2). When evaluating multivariable models using LV ejection fraction instead of heart failure (Supplementary Tables S1, S2) and eGFR instead of CKD (Supplementary Table S1), it was found that there were minimal differences in the estimation of the effect of ACEIs/ARBs.

**Table 2:** Multivariable adjusted 2-year outcomes in patients treated with ACEIs/ARBs vs. those not treated with ACEIs/ARBs.

Variables	HR and *p*-value in the univariable model	HR	95% CI for HR		*p*-Value
			Lower	Upper	
ACEIs/ARBs	0.44, *p* = 0.009	0.51	0.28	0.95	0.034
Sex male	1.61, *p* = 0.147	1.68	0.86	3.25	0.125
Chronic kidney disease	2.19, *p* = 0.012	1.72	0.91	3.25	0.095
Heart failure	3.10, *p* = 0.0002	2.75	1.48	5.13	0.001
Age	1.00, *p* = 0.948	1.00	0.97	1.05	0.696

When ACEIs and ARBs were evaluated individually (Supplementary Table S3), ARBs and ACEIs showed distinct benefits over other antihypertensive medications, with ARBs showing slightly better potential benefits over ACEIs in the reduction of unadjusted cardiovascular mortality (Supplementary Figure S1, Supplementary Table S4). Because of the low number of events in the three arms, we did not make adjustment for confounding variables.

### 3.2. Propensity score–matched cohort analysis

**Figure 2:**
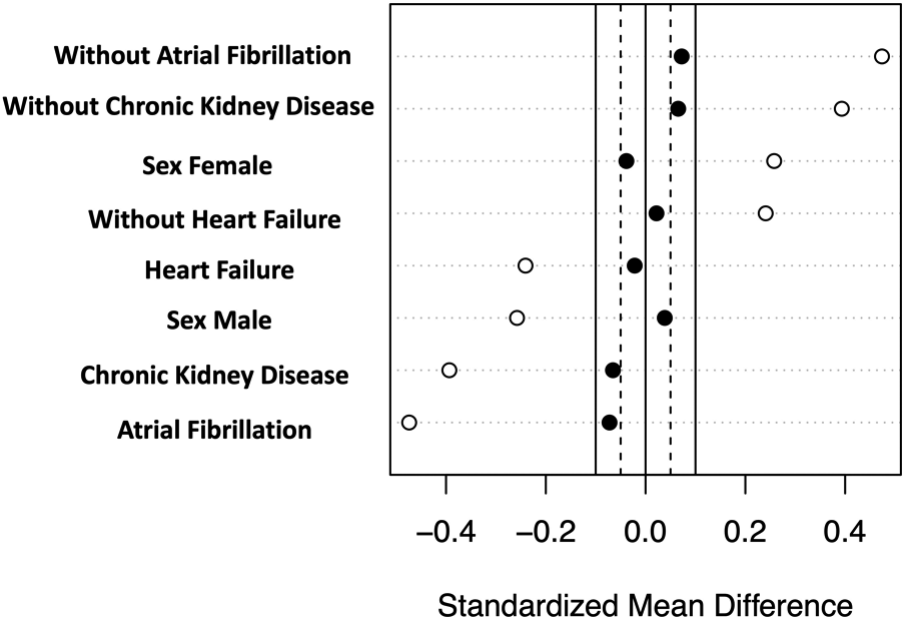
Love plot of standardized mean differences of the propensity score–matched cohort.

**Figure 3:**
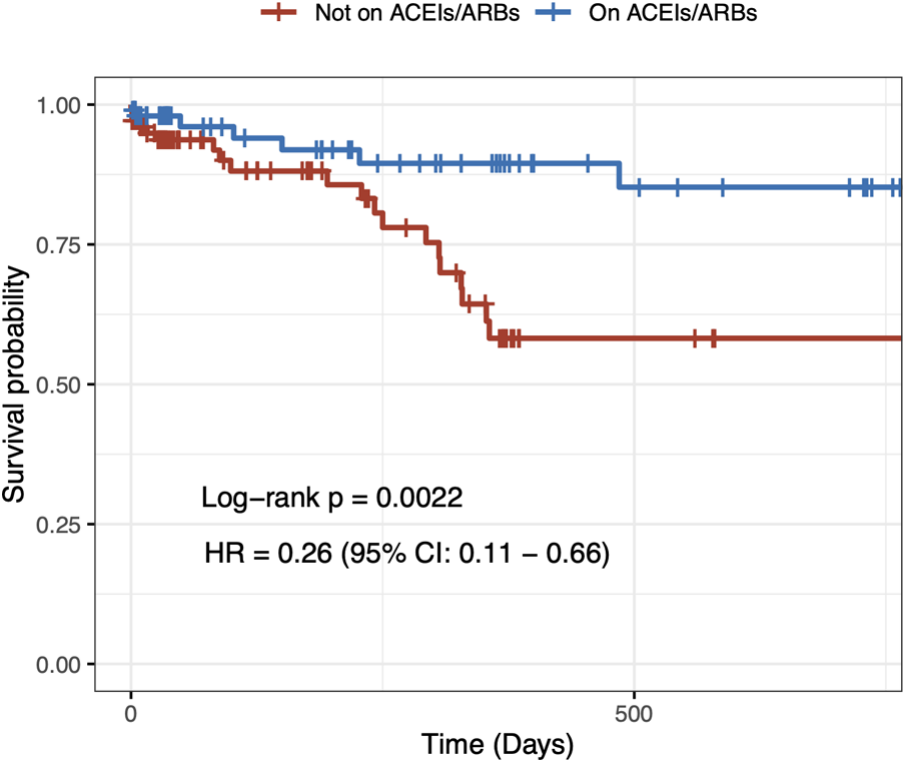
Two-year cardiovascular mortality in the propensity score–matched population.

In order to address the potential selection bias associated with baseline treatment using ACEIs/ARBs, we conducted a propensity score–matched cohort analysis. This cohort consisted of 210 patients, with 105 patients receiving ACEI/ARB therapy at baseline and an equal number of 105 patients who did not receive the therapy at baseline. Importantly, baseline clinical characteristics were found to be comparable between the matched groups, further minimizing any potential differences in the baseline profiles (Table 3, Figure 2). In the propensity score–matched cohort, baseline treatment with ACEIs/ARBs was associated with a decreased risk of cardiovascular mortality (HR = 0.26, 95% CI: 0.11–0.66, *p* = 0.004) (Figure 3).

**Table 3:** Baseline characteristics of a propensity score–matched cohort.

Variables	Patients not on ACEIs/ARBs (*N* = 105) *n*, %	Patients on ACEIs/ARBs (*N* = 105) *n*, %	*p*-Value
Sex male (N/Y)	73 (69.5)	75 (71.4)	0.880
Age (mean ± SD)	83.48 ± 7.83	83.51 ± 5.91	0.968
Weight (kg; mean ± SD)	71.24 ± 17.10	70.39 ± 16.41	0.724
Body mass index (kg/m^2^; mean ± SD)	27.49 ± 6.47	27.62 ± 5.74	0.875
Systolic blood pressure (mmHg; mean ± SD)	128.36 ± 20.05	133.35 ± 20.95	0.079
Diastolic blood pressure (mmHg; mean ± SD)	70.92 ± 10.65	71.98 ± 10.72	0.474
Hemoglobin (g; mean ± SD)	13.00 ± 2.00	12.71 ± 1.80	0.791
Ejection fraction (mean ± SD)	53.80 ± 11.93	54.41 ± 11.14	0.701
Relative wall thickness (mean ± SD)	0.49 ± 0.10	0.49 ± 0.13	0.694
Aortic valve area (cm^2^)	0.46 ± 0.35	0.40 ± 0.32	0.253
Heart failure (N/Y)	38 (36.2)	37 (35.2)	1.000
Chronic kidney disease (N/Y)	45 (42.9)	42 (40.0)	0.779
Atrial fibrillation (N/Y)	40 (38.1)	37 (35.2)	0.775
Diabetes (N/Y)	33 (31.4)	31 (29.5)	0.881
Dyslipidemia (N/Y)	70 (66.7)	71 (67.6)	1.000
Smoking (N/Y)	7 (6.7)	6 (5.7)	1.000
Coronary artery disease (N/Y)	48 (45.7)	44 (41.9)	0.676
Peripheral artery disease (N/Y)	43 (41.0)	51 (48.6)	0.331
Chronic obstructive pulmonary disease (N/Y)	33 (31.4)	30 (28.6)	0.763
Beta blockers (N/Y)	72 (68.6)	69 (65.7)	0.769
Alpha blockers (N/Y)	13 (12.4)	9 (8.6)	0.499
Diuretics (N/Y)	71 (67.6)	76 (72.4)	0.547
Calcium channel blockers (N/Y)	15 (14.3)	32 (30.5)	0.008
Antiplatelet therapy (N/Y)	12 (11.4)	13 (12.4)	0.956
NYHA class II (N/Y)	31 (29.5)	29 (27.6)	0.13
NYHA class III (N/Y)	54 (51.4)	55 (52.4)	0.11
NYHA class IV (N/Y)	20 (19.0)	21 (20.0)	0.47
eGFR (mean ± SD)	57.3 ± 25.0	58.1 ± 24.4	0.82

## 4. Discussion

Our study shows that in hypertensive patients with severe AS undergoing TAVI, baseline treatment with ACEIs/ARBs is associated with a reduced risk of cardiovascular mortality over a 2-year follow-up compared with the use of other antihypertensive medications. The results are also confirmed by the propensity score–matched cohort analysis.

Historically, the use of ACEIs/ARBs in patients with severe AS was considered unsafe and even contraindicated because of concerns about induced severe hypotension caused by vasodilation in the presence of a fixed LV outflow blockade (12). However, this perception was primarily based on theoretical risks and lacked clinical evidence to support it. As shown in more recent studies, there was no increase in the mortality rate in patients with moderate-to-severe AS treated with ARBs, with some studies going as far as to suggest a beneficial effect of ACEIs/ARBs on mortality and the evolution of the natural history of AS (13, 14).

A previous retrospective analysis from the STS/ACC TVT registry (15) showed how treatment with ACEIs/ARBs during hospital discharge in TAVI patients was related to a decreased risk of mortality and hospitalization for heart failure within 1 year, while more recent basic science findings showed an increased benefit of ARBs compared with ACEIs, probably due to their effect on the valve chymase and their ability to block the escape mechanism of RAAS induced by ACEIs (16, 17).

Recently, Fischer-Rasokat et al. analyzed the impact of ACEIs/ARBs on patients who underwent a successful TAVI, demonstrating the beneficial association of ACEIs/ARBs after TAVI and improved survival during follow-up, particularly in high-risk patients, showing a dose-dependent effect (18). The study did not provide any superiority in the effectiveness of ACEIs or ARBs.

In our study, all patients who received ACEIs/ARBs at baseline continued to be treated with these medications at discharge. Therefore, the beneficial effects of baseline treatment with ACEIs/ARBs observed in our study could be attributed, at least partially, to the ongoing use of these medications post discharge. These findings are in line with those of the current literature, which demonstrates that there are significant survival advantages of RAAS inhibition in patients with AS undergoing surgical aortic valve replacement or TAVI (19, 20).

In this study, therapy with ACEIs/ARBs was not only shown to be related to decreased death rates following TAVI but also shown to be safe. However, it is important to acknowledge the potential presence of selection bias in this study, meaning that stable and healthy individuals might have been more likely to receive ACEIs/ARBs compared with their sicker and less stable counterparts who might have been denied such prescriptions. Patients who received ACEIs/ARBs were less likely to have CKD or atrial fibrillation compared with those who did not receive ACEIs/ARBs. Nevertheless, they exhibited similar rates of heart failure and higher rates of chronic coronary syndrome and diabetes. To minimize the impact of this selection bias, we conducted a propensity score–matched cohort analysis.

Calcium channel blockers are also often administered to reasonably stable hypertensive patients and usually avoided in AS patients, because recent studies found a sevenfold increase in the all-cause mortality of patients with AS who were under treatment with calcium channel blockers (21). In contrast to ACEI/ARB medication, which was highly linked to a reduction in cardiovascular mortality, calcium channel blockers showed no significant increase in HR regarding cardiovascular mortality in the present study.

The attenuation of irreversible damage to the LV caused by chronic stress and inappropriate pathological hypertrophy on account of a reduction in the global pressure overload in the LV is one possible explanation for the observed association between ACEI/ARB treatment and favorable outcomes in patients with AS who undergo TAVI. The inhibitory effect of ACEIs/ARBs on hypertrophy and fibrosis is another possible explanation for the link between ACEI/ARB medication and positive outcomes observed in this study (22, 23).

Furthermore, ACEIs/ARBs have been associated with a significant reduction in valve remodeling and valve calcium (24). This effect of ACEIs/ARBs could be explained by their ability to block the RAAS pathway, which, at the valve level, leads to the blocking of the chymase, which prevents inflammation and consequent progressive valve fibro-calcification (16). This pathway seems to be influenced more by ARBs than by ACEIs (16, 24). Although it does not have the ability to detect the presence of any differences in the class of ACEIs/ARBs, if we take into consideration the very low number of events, we can conclude that the data presented in Supplementary Table S4 and Supplementary Figure S1 are at the moment only suggestive of ARBs compared with ACEIs with regard to reduced cardiovascular mortality.

Because the present study is retrospective in nature, it is important to note that further validation through prospective and well-designed studies is needed. Randomized trials are essential to ascertain the safety and prognostic benefits of anti-RAAS medication treatment in patients undergoing TAVI. These trials can also help determine the optimal timing of treatment, such as whether it should be administered prior to or after TAVI, or both. The RASTAVI trial (25) is ongoing and it will randomly assign TAVI patients to receive either ramipril or a placebo. The results of such randomized trials will provide valuable insights into the effectiveness and safety of RAAS inhibition in this specific patient population.

## 5. Limitations

Because of its design, this study cannot demonstrate any direct cause–effect relationship, but it is useful to generate hypotheses.

The EffecTAVI registry was not originally designed or adequately powered to specifically evaluate outcomes on the basis of baseline ACEI/ARB therapy. Although our study found a significant association between lower cardiovascular cause mortality and baseline treatment with ACEIs/ARBs, even after adjusting for various factors, it is important to acknowledge that the possibility of the presence of confounding factors exists because of unmeasured variables that may be correlated to ARB treatment at baseline. To address this concern, we conducted a propensity score–matching cohort analysis, which helped minimize the impact of confounding factors.

In addition, it is worth noting that we did not collect any information on the specific dosage of the antihypertensive drugs used prior to TAVI, which could be relevant in evaluating the impact of treatment. This information could provide further insights into the dosage–response relationship and its effect on outcomes.

## 6. Conclusions

This analysis offers insights into the potential benefits of ACEIs/ARBs in patients with severe AS undergoing TAVI and suggests the potential benefits for reducing cardiovascular mortality. However, these findings await confirmation from highly powered outcome trials.

## Data Availability

The raw data supporting the conclusions of this article will be made available by the authors, without undue reservation.
