# Supplementary material for: Renin–angiotensin system inhibitors reduce cardiovascular mortality in hypertensive patients with severe aortic stenosis undergoing transcatheter aortic valve implantation: insights from the EffecTAVI registry
Source: Front Cardiovasc Med. 2023 Aug 24;10:1234368. doi: 10.3389/fcvm.2023.1234368 (PMC10491454; doi:10.3389/fcvm.2023.1234368)
Supplement: Supplementary file 1 [file Datasheet1.docx]

Supplementary Material

Renin–angiotensin system inhibitors reduce cardiovascular mortality in hypertensive patients with severe aortic stenosis undergoing transcatheter aortic valve implantation: insights from the EffecTAVI registry

Christian Basile^1+^, Costantino Mancusi^1+^, Anna Franzone^1^, Marisa Avvedimento^1^, Luca Bardi^1^, Domenico Angellotti^1^, Domenico Simone Castiello^1^, Andrea Mariani^1^, Rachele Manzo^1^, Nicola De Luca^1^, Plinio Cirillo^1^, Giovanni De Simone^1^, Giovanni Esposito^1,*^

^1^Department of Advanced Biomedical Sciences, University of Naples “Federico II”, Naples, Italy

^+^ These authors contributed equally to this work and share first authorship

***Correspondence:**Prof. Giovanni Esposito

Department of Advanced Biomedical Sciences

University of Naples Federico II

Via Pansini, 5 – 80131 Napoli

e-mail: espogiov@unina.it

tel/fax: +390817461111

# Supplementary Figures and Tables

## Supplementary Tables

**Supplementary Table 1:** Multivariable adjusted 2-year outcomes in patients treated vs. not treated with ACEIs/ARBs.

| *Variables* | *HR* | *95,0% CI for HR* | | *p* |
| --- | --- | --- | --- | --- |
|  |  | *Lower* | *Upper* |  |
| ACEIs/ARBs | 0.53 | 0.28 | 0.99 | 0.047 |
| Sex Male | 1.45 | 0.72 | 2.91 | 0.296 |
| eGFR | 1.03 | 1.01 | 1.035 | 0.002 |
| LV ejection fraction | 0.98 | 0.96 | 1.01 | 0.42 |
| Age | 0.99 | 0.95 | 1.03 | 0.841 |

**Supplementary Table 2:** Multivariable adjusted 2-year outcomes in patients treated vs. not treated with ACEIs/ARBs.

| *Variables* | *HR* | *95,0% CI for HR* | | *p* |
| --- | --- | --- | --- | --- |
|  |  | *Lower* | *Upper* |  |
| ACEIs/ARBs | 0.49 | 0.27 | 0.91 | 0.023 |
| Sex Male | 1.71 | 0.86 | 3.39 | 0.123 |
| CKD | 2.13 | 1.15 | 3.97 | 0.016 |
| LV ejection fraction | 0.99 | 0.96 | 1.02 | 0.455 |
| Age | 1.00 | 0.96 | 1.05 | 0.978 |

**Supplementary Table 3:** Baseline population characteristics, based on ARBs, ACEi or other anti-hypertensive drugs.

| Variables | ARBs (N=110) n, % | ACEIs (N=112) n, % | Other anti-hypertensive medications (N=105) n, % |
| --- | --- | --- | --- |
| Sex female (N/Y) | 69 (62.7) | 57 (50.9) **^*^** | 73 (69.5) **^*^** |
| Age (mean ± SD) | 82.25 ± 6.9 | 82.47 ± 6.25 | 83.48 ± 7.83 |
| Weight (kg; mean ± SD) | 72 ± 15 | 71 ± 15 | 71 ± 17 |
| Body Mass Index  (kg/m^2^; mean ± SD) | 28 ± 6 | 27 ± 5 | 27 ± 6 |
| Systolic Blood Pressure (mmHg; mean ± SD) | 135 ± 18 **^+^** | 133 ± 21 | 128 ± 20 **^+^** |
| Diastolic Blood Pressure (mmHg; mean ± SD) | 73 ± 12 | 71 ± 10 | 71 ± 11 |
| Hemoglobin (g; mean ± SD) | 12 ± 2 | 13 ± 1 | 13 ± 2 |
| Ejection Fraction (mean ± SD) | 55.7 ± 10.9 | 53.1 ± 11.8 | 53.8 ± 11.9 |
| Relative Wall Thickness (mean ± SD) | 0.49 ± 0.11 | 0.47 ± 0.11 | 0.49 ± 0.10 |
| Aortic valve area (cm^2^) | 0.43 ± 0.34 | 0.38 ± 0.34 | 0.46 ± 0.35 |
| Diabetes (N/Y) | 47 (42.7) | 39 (34.8) | 33 (31.4) |
| Dyslipidemia (N/Y) | 74 (67.3) | 79 (70.5) | 70 (66.7) |
| Smoking (N/Y) | 6 (5.5) | 14 (12.5) | 7 (6.7) |
| Coronary Artery Disease (N/Y) | 45 (40.9) | 52 (46.4) | 48 (45.7) |
| Peripheral Artery Disease (N/Y) | 55 (50) | 49 (43.8) | 43 (41) |
| Chronic Obstructive Pulmonary Disease (N/Y) | 32 (29.1) | 35 (31.3) | 33 (31.4) |
| Heart Failure (N/Y) | 26 (23.6) | 31 (27.7) | 38 (36.2) |
| Chronic Kidney Disease (N/Y) | 27 (24.5) **^+^** | 30 (26.8) **^*^** | 45 (42.9) **^+*^** |
| Atrial Fibrillation (N/Y) | 22 (20) **^+^** | 21 (18.8) **^*^** | 40 (38.1) **^+*^** |
| Calcium Channel Blockers (N/Y) | 26 (23.6) | 33 (29.5) **^*^** | 15 (14.3) **^*^** |
| Anti-platelet therapy (N/Y) | 70 (63.6) | 82 (73.2) **^*^** | 57 (54.3) **^*^** |
| Beta-blockers (N/Y) | 71 (64.5) | 79 (70.5) | 72 (68.6) |
| Alpha-blockers (N/Y) | 14 (12.7) | 10 (8.9) | 13 (12.4) |
| Diuretics | 69 (62.7) | 77 (68.8) | 71 (67.6) |
| Cardiovascular mortality (N/Y) | 9 (8.2) **^+^** | 14 (12.5) **^*^** | 20 (19) **^+*^** |

* p<0.05 ACEIs VS Not on ACEIs

+ p<0.05 ARBs VS Not on ACEIs

**Supplementary Table 4:** Univariate 2-year outcomes in patients treated with ACEIs or ARBs vs. patients treated with other anti-hypertensive medications.

| *Variables* | *HR* | *95,0% CI for HR* | | *p* |
| --- | --- | --- | --- | --- |
|  |  | *Lower* | *Upper* |  |
| ACEIs | 0.58 | 0.40 | 0.86 | 0.018 |
| ARBs | 0.35 | 0.15 | 0.78 | 0.011 |

## Supplementary Figures

**Supplementary Figure 1:** Two-year unadjusted cardiovascular mortality. Crude Kaplan–Meier survival curves according to angiotensin-converting enzyme inhibitors, angiotensin II receptor blockers or other anti-hypertensive medications at baseline in hypertensive patients undergoing transcatheter aortic valve implantation.
